# The minimal important difference and smallest detectable change of the Oxford elbow score, *Quick* disabilities of the arm shoulder and hand and single assessment numeric evaluation used for elbow trauma

**DOI:** 10.1016/j.jseint.2024.02.011

**Published:** 2024-03-22

**Authors:** Johan Wänström, Eythor Ö. Jonsson, Hanna Björnsson Hallgren, Albin Jorméus, Lars Adolfsson

**Affiliations:** a Department of Orthopaedics, Helsingborg Hospital, Helsingborg, Sweden; b Division of Orthopaedic Surgery, Department of Biomedical and Clinical Sciences, Linkoping University, Linkoping, Sweden; c Department of Orthopaedics, Institute of Clinical Sciences, The Sahlgrenska Academy, University of Gothenburg, Gothenburg, Sweden; d Department of Orthopaedics, Sahlgrenska University Hospital, Mölndal, Sweden

**Keywords:** Minimal important difference, Smallest detectable change, Oxford elbow score, Quick disabilities of the arm shoulder and hand, Single assessment numeric evaluation, Elbow, TRAUMA, Patient-reported outcome measures

## Abstract

**Background:**

The Minimal Important Difference (MID) and Smallest Detectable Change (SDC) are methods used to identify the smallest changes in Patient-Reported Outcome Measures (PROMs) that are of relevance to the patients. Data on these parameters is, however, limited for elbow conditions including traumatic injuries. The aim of this study was, therefore, to estimate the MID and SDC for three commonly used PROMs after elbow trauma; the Oxford Elbow Score (OES), *Quick* Disabilities of the Arm Shoulder and Hand (*Quick*DASH) and Single Assessment Numeric Evaluation (SANE).

**Methods:**

One hundred patients, 67 females, aged ≥18 years (mean age 52.4 years (standard deviation, 18.2)), who had sustained a fracture, tendon rupture or dislocation affecting the elbow, completed the OES, *Quick*DASH, and SANE 3-5 months after injury (T1) and again after a minimum of 3 weeks (T2). A transition item with a 7-level scale, enquiring about the situation with the elbow, was also completed at T1 and T2. The difference in scores between T1 and T2 was calculated (change scores). The MID was assessed using the mean change method; a response of “slightly better” or “slightly worse” was defined as being a clinically significant change. The SDC was estimated by calculating the standard error of measurement based on 2 administrations (1- to 3-week interval) of PROMs in a separate group of patients who had sustained an elbow injury 1- 2 years previously.

**Results:**

The most common diagnosis was fracture of the proximal radius (n = 33). Eighteen patients responded slightly better and 5 slightly worse on the transition item and had mean change scores of 7.9 (9.3) for the OES and −7.4 (11.4) for the *Quick*DASH. Assessment of SDC was based on 56 patients having sustained an elbow injury between September 2019 and October 2020. The SDC was: 12.1 for the OES, 11.4 for the *Quick*DASH, and 1.94 for the SANE.

**Conclusion:**

Change scores need to exceed 12.1 points for the OES, 11.4 points for the *Quick*DASH, and 1.94 points for the SANE in order to measure change with clinical relevance and not due to measurement errors.

Trauma is one of the most common conditions affecting the elbow. Patient-Reported Outcome Measures (PROMs) are increasingly used to assess the outcome of elbow trauma and related treatments. The interpretation of differences in scores of PROMs between groups is aided by estimates of the Minimal Important Difference (MID) and Smallest Detectable Change (SDC). The concept of MID was initially defined in 1989 by Jaersecke et al^11^ and it is not considered a measurement property of an instrument but an important characteristic.^15^ MID can be defined as the smallest measured change in a score which the patient perceives as important.^6^^,^^11^ MID is affected by diagnosis and a particular estimate is specific to the diagnoses in the study population it was determined for. While the MID assesses which difference in scores patients find important, the SDC assesses statistical limitations when comparing scores. SDC has been defined as the smallest change that can be detected by an instrument, beyond measurement error.^23^

Limited data is available on the MID and SDC of PROMs used for assessing patients after elbow trauma and related treatment. The Oxford Elbow Score (OES) is the only currently available PROM for elbow conditions that has been validated with high quality methodology.^21^ The *Quick* Disabilities of the Arm Shoulder and Hand (*Quick*DASH) and Single Assessment Numeric Evaluation (SANE) are two other available PROMs; both attractive as they consist of a low number of items. The MID of the OES has been assessed in two previous studies. Dawson et al estimated the MID of the domains of the OES as follows: Function ≈ 10, Pain ≈ 18 and Social-psychological ≈ 18.^4^ However, no patients with fresh trauma were included in this study. In a study specifically assessing the MID after elbow dislocations, Iordens et al derived the following values: total OES score 8.2, Function 5.6, Pain 11.4 and Social-Psychological 11.7. They noted that a ceiling effect might have affected some domains and led to lower values.^10^ Both of these studies used the OES with a 4-week recall period. Recently a version of OES with a 7-day recall period demonstrated good measurement properties, supporting its validity and use.^12^ The SDC of the OES and its domains has been reported in several studies using a 4-week recall period but not with the 7-day recall period.^4^^,^^8^^,^^10^^,^^18^

MID for *Quick*DASH has previously been estimated for elbow trauma by Randall DJ et al to be between 5.3 and 11.7 in a retrospective cohort study.^19^

To our knowledge there have not been any estimations of MID for the SANE.

The aim of this study was to estimate the MID and SDC for elbow trauma using the OES with a 7-day recall period, *Quick*DASH, and SANE. To the best of our knowledge, neither the MID or SDC of the OES have previously been assessed for elbow trauma.

## Materials and methods

### Study design and settings

This is a cohort study based on prospectively collected data. Patients were recruited at 3 hospitals in Sweden: Linkoping University Hospital, Linkoping; Sahlgrenska University Hospital, Gothenburg and Helsingborg Hospital, Helsingborg (a county hospital).

To calculate SDC, a separate group of 56 patients having sustained an elbow trauma 1-2 years earlier was identified from hospital records. All were in a steady state after having completed their respective treatments since at least 1 year.

### Eligibility criteria

Inclusion criteria were individuals over 18 years of age with and a fracture, tendon rupture or dislocation affecting the elbow. Exclusion criteria were concurrent injuries significantly affecting upper extremity function or an inability to participate in assessments (eg, cognitive impairment or insufficient understanding of the Swedish language).

### Outcome measures

The OES is a PROM developed to assess outcomes after surgery of the elbow.^5^ The OES consists of 12 items grouped into 3 domains: *elbow function, pain and social-psychological* aspects. Each item has five response options that are scored from 0 to 4, with lower numbers representing greater impairment. The results are summarized by dividing the total score of the items by the maximum possible score, and then multiplying by 100. In this study the recently introduced 7-day version of OES was used in which patients were asked to respond to items based on their experiences during the past week, as opposed to the previous 4 weeks in the original version.^12^

The *Quick*DASH is an 11-item PROM that is a shortened version of the Disabilities of the Arm Shoulder and Hand (DASH), which is intended to assess physical function in populations with upper extremity musculoskeletal conditions.^2^^,^^9^ The *Quick*DASH is scored from 0-100 with higher scores indicating worse function. A Swedish language version of the *Quick*DASH has previously been found to be a valid outcome measure for upper extremity disorders.^7^

The term “function” was incorporated into the SANE question:*” How would you rate your current elbow function compared with a completely normal elbow?”* Responses were recorded on an 11-step Numerical Rating Scale.^1^^,^^22^

### Data collection

Patients having sustained a recent elbow trauma responded to the OES, *Quick*DASH, and SANE at two different time points: 3-5 months after injury (termed T1) and again at least 3 weeks later (termed T2). Patients completed questionnaires when visiting outpatient clinics or received them by regular mail and returned completed questionnaires by post.

Patients responded to a transition item at T2. The transition item at T2 was phrased in the following way:” How *is your elbow now compared with the last time you completed these questionnaires?* Answers were recorded on a 7-point global assessment of change scale with the following response options: *Worse than ever, Much worse, Slightly worse, No difference, Slightly better, Much better, and Recovered.*

### Data management and statistical analysis

The data were computerized with Filemaker Pro version 15 (Claris International, Santa Clara, CA, USA). OES questionnaires with any missing item and *Quick*DASH questionnaires with 1 or more missing items were excluded.^2^ The data were analyzed in SPSS version 26 (IBM, Armonk, NY, USA). Continuous variables were presented as mean and standard deviation, while categorical variables were presented as a number (n) and a percentage (%). The results of statistical tests were considered significant when the *P* value was less than .05.

**Figure 1:**
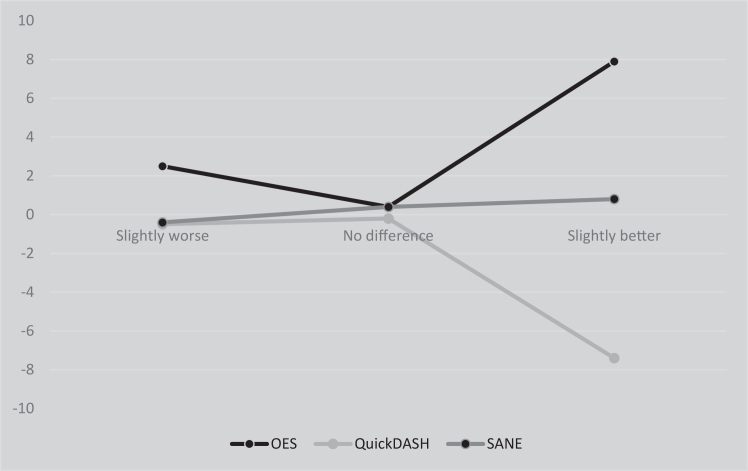
Difference in means for transition items: “Slightly worse”, “No difference” and “Slightly better”. *OES*, Oxford shoulder score; *QuickDASH*, quick disabilities of the arm shoulder and hand score; *SANE*, single assessment numeric evaluation.

Changes scores for the clinical rating systems (OES, *Quick*DASH, and SANE) were calculated between T1 and T2 by subtracting the score at T1 from the score at T2 for each patient. MID was assessed for PROMs which had correlations (Pearson’s) of >0.28 with the transition.^20^ The mean change scores of the clinical rating systems were calculated for each of the 7 response options of the transition items. The primary analysis of MID was according to the mean change method taken to be the mean change score for the clinical rating systems for patients that responded “slightly better” or “slightly worse” on the transition item (Fig. 1).^17^

SDC was determined by repeated observations in a separate group of 56 patients in a steady state, 1-2 years after elbow trauma. Patients were identified in hospital records and received questionnaires by mail. They completed the OES, *Quick*DASH, and SANE on two occasions, 1 week apart (maximum 3 weeks). The SDC was calculated based on the S*tandard Error of Measurement* (SEM) in the following way: $SDC=SEM\times 1.96\times \sqrt{2}$.^24^ The SEM quantifies the precision of individual scores, being the standard deviation of repeated measurements in one patient.^14^^,^^25^ The SEM has the same units as the measurement of interest. The SEM was calculated from the square root of the error variance between the measurements derived from calculations of Intraclass Correlation Coefficients (ICCs). Calculations of ICCs were based on a 2-way, mixed-effects, and absolute agreement model.^13^

### Ethical issues

The study was approved by the Swedish Ethical review authority (Dnr 2020-00749 and Dnr 2020-04709). All patients signed an informed consent for participation in the study.

## Results

### Demographic, injury, and treatment-related variables

Between March 2020 and April 2022, the study enrolled 100 patients. The mean overall age of the patients was 52 years (18.2) with 67 being females. Injury-related characteristics are presented in Table I. 70 patients underwent surgical treatment with a mean of 6.5 (4.8) days passing between injury and surgery.

**Table I:** Injury-related data.

Characteristic	n (%)
Injured side	
Right	39 (39)
Left	61 (61)
Dominant side injured	65 (65)
Injury mechanism	
Ground level fall	53 (53)
Bicycle accident	17 (17)
Fall from height	9 (9)
Sports	8 (8)
Other	13 (13)
Low energy injury mechanism	85 (85)
Injury type	
Proximal radius fracture	33 (33)
Olcranon fracture	13 (13)
Terrible triad	12 (12)
Elbow dislocation	11 (11)
Monteggia fracture	9 (9)
Distal humeral fracture	8 (8)
Other	14 (14)

### Change scores (T1 to T2) of the OES, *Quick*DASH, and SANE and correlation with transition items

**Figure 2:**
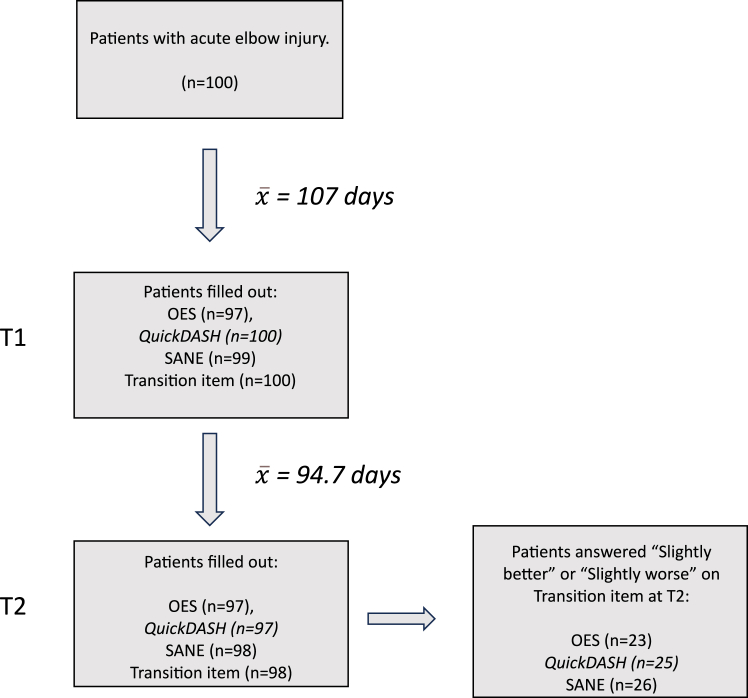
Flowchart illustrating patient flow. *OES*, Oxford shoulder score; *QuickDASH*, quick disabilities of the arm shoulder and hand score; *SANE*, single assessment numeric evaluation; *T1*, completed 3-5 months after injury; *T2*, completed again after a minimum of 3 weeks.

The number of patients with missing values for the OES was (n): T1 (3) and T2 (3). The number of patients with one missing value for the *Quick*DASH for each of the two time periods was (n): T1 (0) and T2 (3). Across the two time periods, the mean number of days between injury and completing questionnaires was: T1, 107 (18.9); T2, 202 (54.7). The mean number of days between completing T1 and T2 was 94.7 (50.4) (Fig. 2). There was an overall improvement between T1 and T2 in the results of the OES total score (encompassing all three OES domains), *Quick*DASH, and SANE (Table II). The differences in the mean values of the OES (n = 97), *Quick*DASH (n = 97) and SANE (n = 97) at T1 compared to T2 were statistically significant, all *P* values < .001.

**Table II:** Scores at T1 and T2 and change scores between these timepoint for the OES, *Quick*DASH, and SANE.

	T1∗		T2†		Change score T1/T2	
	n	Mean (SD)	n	Mean (SD)	n	Mean (SD)
OES						
Total score	97	73.9 (21.1)	97	81.8 (19.3)	97	7.8 (10.3)
Elbow function	97	78.7 (20.1)	97	86.9 (16.5)	97	8.2 (12.2)
Pain	97	77.5 (21.0)	97	83.6 (20.3)	97	6.1 (11.8)
Social-psychological	97	65.5 (27.4)	97	74.7 (24.9)	97	9.1 (16.3)
*Quick*DASH	100	23.5 (21.4)	97	17.0 (18.7)	97	−7.2 (11.1)
SANE	99	6.8 (2.2)	98	7.5 (1.9)	97	0.7 (1.4)

*SD*, standard deviation; *OES*, Oxford shoulder score; *QuickDASH*, quick disabilities of the arm shoulder and hand score; *SANE*, single assessment numeric evaluation; *T1*, completed 3-5 months after injury; *T2*, completed again after a minimum of 3 weeks.

∗ T1, a period of 7 days after 3-5 months from injury.

† T2, a period of 7 days, 1.5-12 months after T3.

The magnitude of correlation coefficients for correlation between transition items and change scores of the OES, *Quick*DASH, and SANE were in the range 0.23-0.43 (Table III).

**Table III:** Correlation between the transition item and change scores of the OES (total score, elbow function, pain, social-psychological), *Quick*DASH, and SANE.

	OES				*Quick* DASH	SANE
	Total score	Function	Pain	Social-psychological		
Transition item	0.43	0.23	0.4	0.36	−0.33	0.29

*OES*, Oxford shoulder score; *QuickDASH*, quick disabilities of the arm shoulder and hand score; *SANE*, single assessment numeric evaluation.

Using the mean change method, estimates of MID were as follows: OES total score 7.9 (9.3), OES function 9.4 (11.6), OES pain 5.9 (7.6), OES social-psychological 8.3 (15.7), *Quick*DASH −7.4 (11.4) and SANE 0.8 (1.4), (Table IV).

**Table IV:** Change scores the OES, Quick-DASH and SANE.

Transition item	OES		QuickDASH		SANE	
	Total					
	n	Mean (SD)	n	Mean (SD)	n	Mean (SD)
Worst then ever	0		0		0	
Much worse	2	−22.9 (14.7)	2	16.8 (5.1)	1	−4 (-)
Slightly worse	5	2.5 (5.8)	5	−0.5 (9.8)	5	−0.4 (0.9)
No difference	14	0.4 (4.2)	13	−0.2 (5.3)	13	0.4 (1.0)
Slightly better	18	7.9 (9.3)	20	−7.4 (11.4)	21	0.8 (1.4)
Much better	44	12.8 (8.9)	44	−12.2 (10.8)	43	1.1 (1.3)
Recovered	14	5.8 (7.7)	13	−3.0 (3.5)	14	0.6 (1.3)

*SD*, standard deviation; *OES*, Oxford shoulder score; *QuickDASH*, quick disabilities of the arm shoulder and hand score; *SANE*, single assessment numeric evaluation.

The slightly better and slightly worse group (in italics) was used for MIC, calculation in this study.

#### Smallest detectable change (SDC)

The mean duration of time between the administrations was 9.1 days (3.2). The calculated SDC scores were as follows: OES total score (12.1), *Quick*DASH (11.4) and SANE (1.94) (Table V).

**Table V:** Assessment of smallest detectable change for the OES, *Quick*DASH, and SANE.

	SEM	SDC
OES		
Total score	1.0	12.1
Elbow function	1.2	14.9
Pain	1.2	20.8
Social-psychological	6.4	17.7
*Quick*DASH	4.1	11.4
SANE	0.7	1.94

*OES*, Oxford shoulder score; *QuickDASH*, quick disabilities of the arm shoulder and hand score; *SANE*, single assessment numeric evaluation; *SDC*, smallest detectable change; *SEM*, standard error of measurement; *SANE*, single assessment numeric evaluation.

## Discussion

The present study found the SDC change to be 12.1 and the MID 7.9 for the OES. For *Quick*DASH the SDC was 11.4 and the MID 7.4. Additionally, the SDC was 1.94 and the MID was 0.8 for the SANE when “slightly better” was used as the change score. When both “slightly better” and “slightly worse” were included, the MID values were 6.0 for the OES, 5.4 for *Quick*DASH, and 0.7 for SANE (Table VI).

**Table VI:** Change scores between T3 and T4 of the OES, QuickDASH, and SANE when both “Slightly worse” and “Slightly better” are included.

	n	Mean (SD)
OES		
Total score	23	6.0 (9.0)
*Quick*DASH	25	−5.4 (12.4)
SANE	26	0.7 (1.4)

*SD*, standard deviation; *OES*, Oxford shoulder score; *QuickDASH*, quick disabilities of the arm shoulder and hand score; *SANE*, single assessment numeric evaluation.

Calculated MIDs have exhibited a broad range of values for the same PROM.^3^ This variance may in part be due to differences in methods and definitions used in the respective studies but even with more standardized methodology a range of values for MID estimates for a particular PROM is expected to remain.^5^^,^^10^ Factors such as diagnosis, disease severity, time point and demographic factors are likely to influence MID estimates.^16^ MID estimates remain just that, an estimate that can be useful when interpreting differences between groups or planning groups sizes for studies but it is dubious to assume that there will be one true MID for a particular PROM. Although it is important to choose the most appropriate estimate based on factors such as diagnosis and demographic variables, for the particular situation a range of relevant values will remain. One major reason is that the particular transition item and response scale used are likely to affect the estimate of the MID and there is no obvious correct choice.

Measurement error due to random variation must also be considered when interpreting changes or discrepancies in clinical rating systems scores. The SDC is a measure of random variation.^6^ A difference in results of a clinical rating system between two time points (change value) needs to be higher than the SDC in order to be considered real and not merely due to chance alone. To be able to determine whether a change score for an individual patient is clinically important the SDC should not exceed the MID. Failure to meet this condition means, we cannot be 95% sure that this change is not due to measurement error. In the present study, the SDC was larger than the MID but the difference was rather small. It appears probable that the PROMs used are suitable for clinical practice. A change score larger than MID but smaller than SDC should be interpreted with care, since it could also be due to chance. Consequently, summarizing the findings of the study of the PROMs analyzed in this study the SDC appears a more relevant estimate to reliably detect a minimal change of importance to the patient.

When both “slightly worse” and “slightly better” were considered in the change score, the MID was lower than when “slightly better” was used as the change score alone and still lower than the SDC. Although there were only 5 patients who responded “slightly worse” at the transition item, their mean score was lower for the OES, QuickDASH, and for SANE compared to patients who responded “slightly better”. The lower MID and mean scores may indicate that a smaller disparity in OES, QuickDASH, and SANE scores are perceived as important by the patient.

Five of the patients who scored zero on *Quick*DASH and 48 on OES at T2, ie, according to these PROMs were fully recovered, scored only 9 on SANE at T2. After calling these patients up, 2 said they were fully recovered but 3 still had problems with the elbow. Although there were only 3 patients, it is interesting that the problems in these patients’ elbows were not detected by either *Quick*DASH or OES.

## Conclusion

Change scores need to exceed 12.1 points for the OES, 11.4 points for the *Quick*DASH, and 1.94 points for the SANE to be clinically relevant and not due to measurement error.

## Acknowledgment

The authors thank the upper extremity colleagues at Linköping University Hospital for help with data collection. Terez Zara and Patricia Larsson for help with administrative work and Stig and Ragna Gorthons’ foundation and ALF Grants, Region Östergötland for funding.

## Disclaimers:

Funding: Mr. Wänström received a grant from the Stig and Ragna Gorthonś Fund, Helsingborg, Sweden. Dr. Jonsson received a grant from The Gothenburg Society of Medicine. Assoc. Prof. Hallgren was supported by the Sweden 10.13039/100010805Medical Research Council of Southeast Sweden (project number 30522028 ALF: Tid 551-53150).

Conflicts of interest: The authors, their immediate families, and any research foundation with which they are affiliated have not received any financial payments or other benefits from any commercial entity related to the subject of this article.
